# The *APOA5 *Trp19 allele is associated with metabolic syndrome via its association with plasma triglycerides

**DOI:** 10.1186/1471-2350-9-84

**Published:** 2008-09-12

**Authors:** Jean Dallongeville, Dominique Cottel, Aline Wagner, Pierre Ducimetière, Jean-Bernard Ruidavets, Dominique Arveiler, Annie Bingham, Jean Ferrières, Philippe Amouyel, Aline Meirhaeghe

**Affiliations:** 1 Institut Pasteur de Lille, INSERM, U744, Université de Lille 2, Lille, France; 2 EA 1801, Laboratoire d'Epidémiologie et Santé Publique, Université Louis Pasteur, Faculté de Médecine, Strasbourg, France; 3 INSERM, U780, Villejuif, Université Paris-Sud XI, France; 4 INSERM, U558, Toulouse, Faculté de Médecine, Université Paul Sabatier, Toulouse, France

## Abstract

**Background:**

The goal of the present study was to assess the effect of genetic variability at the APOA5/A4/C3/A1 cluster locus on the risk of metabolic syndrome.

**Methods:**

The *APOA5 *Ser19Trp, *APOA5 *-12,238T>C, *APOA4 *Thr347Ser, *APOC3 *-482C>T and *APOC3 *3238C>G (*Sst*I) polymorphisms were analyzed in a representative population sample of 3138 men and women from France, including 932 individuals with metabolic syndrome and 2206 without metabolic syndrome, as defined by the NCEP criteria.

**Results:**

Compared with homozygotes for the common allele, the odds ratio (OR) [95% CI] for metabolic syndrome was 1.30 [1.03–1.66] (*p *= 0.03) for *APOA5 *Trp19 carriers, 0.81 [0.69–0.95] (*p *= 0.01) for *APOA5 *-12,238C carriers and 0.84 [0.70–0.99] (*p *= 0.04) for *APOA4 *Ser347 carriers. Adjustment for plasma triglycerides, (but not for waist girth, HDL, blood pressure or glycemia – the other components of metabolic syndrome) abolished these associations and suggests that triglyceride levels explain the association with metabolic syndrome. There was no association between the *APOC3 *-482C>T or *APOC3 *3238C>G polymorphisms and metabolic syndrome. The decreased risk of metabolic syndrome observed in *APOA5 *-12,238C and *APOA4 *Ser347 carriers merely reflected the fact that the *APOA5 *Trp19 allele was in negative linkage disequilibrium with the common alleles of *APOA5 *-12,238T>C and *APOA4 *Thr347Ser polymorphisms.

**Conclusion:**

The *APOA5 *Trp19 allele increased susceptibility to metabolic syndrome via its impact on plasma triglyceride levels.

## Background

Metabolic syndrome is a complex disease characterized by the ustering of several metabolic disorders [1,2]. Excess body weight, insulin resistance, altered plasma lipid levels & glucose homeostasis and increased blood pressure are the principal components of this cluster. Environmental influences (such as low physical activity and an inappropriate diet) play a major role in the development of metabolic syndrome. Furthermore, familial aggregation of metabolic disorders has been reported and suggests a genetic contribution to the etiology of this syndrome [3-5]. Accordingly, it has been reported that genetic variability at several loci is associated with an increased risk of metabolic syndrome [6-12].

The *APOA5 *gene is located on chromosome 11q23 and codes for a 369 amino-acid protein secreted by the liver [13]. In the plasma, APOA5 is bound to triglyceride-rich and high-density lipoproteins. APOA5 reduces plasma triglyceride levels by inhibiting VLDL-triglyceride production and stimulating LPL-mediated triglyceride hydrolysis [14-18]. *In vitro*, insulin decreases *APOA5 *promoter activity – suggesting that the hormone may affect triglyceride levels via an interaction with APOA5 [19]. Given its role in triglyceride metabolism and its interaction with insulin, the *APOA5 *gene is a candidate gene for metabolic syndrome.

Several common single nucleotide polymorphisms (SNPs) have been described in the *APOA5 *gene locus; these include a T>C substitution at -12,238 (SNP4), a T>C substitution at -1131 (SNP3; rs662799) and a C>G mutation at the first base of codon 19 (rs3135506; c.56C>G) that changes a serine (Ser) residue into a tryptophan (Trp) [20]. An association between the *APOA5 *Ser19Trp polymorphism and plasma triglycerides has been reported in several population samples [13,20-23]. In contrast, an association between the *APOA5 *-12,238T>C polymorphism and plasma lipid variables has not been consistently observed. Together with *APOA4*, *APOC3 *and *APOA1*, the *APOA5 *gene lies within an expanded gene cluster [13,24]. Strong linkage disequilibrium (LD) has been detected for SNPs in this cluster [24]. The *APOA5 *Ser19Trp and *APOC3 *3238C>G SNPs (*Sst*1; rs5128; c.386C>G) both tag common haplotypes in this cluster – haplotypes that may have independent effects on plasma triglycerides.

The goal of the present study was to assess the relative contribution of SNPs in the APOA5/A4/C3/A1 locus to the risk of metabolic syndrome by analyzing a limited number of representative SNPs within the cluster and a large population-based sample from 3 areas of France.

## Methods

### Subjects

Participants were recruited as part of the WHO-MONICA population survey conducted from 1995 to 1997 in three different parts of France: the Lille Urban Community in northern France (n = 1168), the Bas-Rhin county in eastern France (n = 1117) and the Haute-Garonne county in southern France (n = 1176). The protocol was approved by the appropriate independent ethics committee in each center. Subjects (aged 35–64) were randomly selected from electoral rolls after stratification by town size, gender and age in order to obtain 200 participants for each gender and each 10-year age group (WHO-MONICA Project protocol) [25]. A total of 1746 men and 1715 women completed the recruitment procedure. Subsequently, 323 subjects were excluded due to missing data on at least one criterion for metabolic syndrome.

After providing written informed consent, participants filled out a standard questionnaire and physical measurements were taken by a specially trained nurse. The questionnaire covered questions on socioeconomic factors, physical activity, alcohol consumption, smoking status, personal & family medical history and current medications taken (if any). The level of leisure-time physical activity during was categorized as follows: none, light (light physical activity almost every week), intense (at least 20 minutes of intense physical activity more than once a week). In terms of smoking exposure, subjects were categorized as never-smokers, former smokers and current smokers (i.e. subjects reporting at least one cigarette per day). Total alcohol intake was expressed as the sum of ml alcohol per week from wine, beer, cider and spirits. Alcohol consumption was categorized in 4 Q1-Q4 classes for men [≤100 ml/week; 100–250 ml/week; 250–500 ml/week; >500 ml/week] and for women [0 ml/week; 0–60 ml/week; 60–190 ml/week; > 190 ml/week]. Educational level was assessed by counting the number of years of schooling and classifying the values into 3 categories: primary, secondary/technical and university. Anthropometric measurements included body weight (rounded to the nearest even decimal), waist girth (at a level midway between the lower rib margin and the iliac crest, to the nearest 0.5 cm) and were performed on subjects in light clothing without shoes. Body mass index was calculated according to the Quetelet equation.

Diabetic subjects were identified by fasting glycemia ≥126 mg/dl or antidiabetic treatment. Abdominal obesity was defined by waist girth ≥102 cm in men and ≥88 cm in women. Metabolic syndrome was defined (according to the NCEP III recommendations [26]) by the presence of at least 3 or more of the following abnormalities: waist girth ≥102 cm in men and ≥88 cm in women, triglycerides ≥ 1.7 mmol/L or treatment with fibrates, HDL-cholesterol <1.04 mmol/L in men and <1.29 mol/L in women, blood pressure ≥ 130/85 mm Hg or treatment with blood pressure-lowering medications and fasting glucose ≥ 6.1 mmol/L or type 2 diabetes. Additional analyses were performed using the International Diabetes Federation (IDF) definition [27] of metabolic syndrome. This definition is based on the same criteria as the NCEP definition except that (i) increased waist girth is a mandatory criteria and (ii) the waist cut-offs are 94 and 80 cm for European men and women, respectively.

### Laboratory methods

A 20 mL blood sample was drawn into a disodium EDTA tube (after the subjects had fasted for at least 10 hours), stored at room temperature and centrifuged within 4 hours. All measurements were performed in a central laboratory (Purpan Hospital, Toulouse, France). Cholesterol and triglyceride levels were measured using enzyme assays (Boehringer Mannheim, Mannheim, Germany). High density lipoprotein (HDL) cholesterol was measured after sodium phosphotungstate/magnesium chloride precipitation (Boehringer Mannheim, Mannheim, Germany). Glucose was measured using the standard glucose hexokinase method (DuPont Dimension, Brussels, Belgium). The *APOA5 *Ser19Trp polymorphism was assessed in an Amplifluor^® ^genotyping system using the following probes: APOA5-F1: 5' GAAGGTGACCAAGTTCATGCTTCCCTCTCCACAGCGTTTTC-3'; APOA5-F2: 5'-GAAGGTCGGAGTCAACGGATTTCCCTCTCCACAGCGTTTTG-3' and APOA5-R: 5'-TGAAGTAGTCCCAGAAGCCTTT-3'. In the sample, 1.6% was undetermined. Likewise, the *APOC3 *3238C>G (*Sst*I) polymorphism was assessed using the following probes: APOC3-F1: 5'-GAAGGTCGGAGTCAACGGATTACCTGCCTATCCATCCTGCC-3', APOC3-F2: 5'-GAAGGTGACCAAGTTCATGCTACCTGCCTATCCATCCTGCG-3' and APOC3-R: 5'-GCACTGAGAATACTGTCCCTTT-3'. In the sample, 3.5% was undetermined. The *APOC3 *-482C>T (rs2854117) polymorphism was also assessed in an Amplifluor^® ^system using the following probes: APOC3-F1: 5'-GAAGGTGACCAAGTTCATGCTAGTAAAGGCACAGAAGACCG-3', APOC3-F2: 5'-GAAGGTCGGAGTCAACGGATTGAGTAAAGGCACAGAAGACCA-3' and APOC3-R: 5'-TGAAACCCAGAGATGGAGGT-3'. In the sample, 3.8% was undetermined. The *APOA4 *Thr347Ser polymorphism was assessed as previously described [28]. The *APOA5 *-12,238T>C polymorphism was genotyped with an RFLP-based method using the following primers: forward 5'-GTGCCTGTCACCACCGTTTG-3' and reverse 5'-ATGCATTAGCCTCTGCTGTTC-3' (generating a forced restriction site for *Hae*III). The 162 bp PCR fragment was cut by *Hae*III into 2 fragments (141 and 21 bp) for the T allele, whereas a single uncut product resulted for the C allele. Products were resolved on 3% agarose gels. For each method, around 10% of the samples were genotyped twice and no discordant genotypes were observed.

### Statistical analyses

General linear models (GLM) and chi-square tests were used to compare the clinical and biological characteristics of subjects with or without metabolic syndrome. Furthermore, GLM and Chi-square tests were used to compare centers in terms of mean values and the distributions of variables and genotypes, respectively. A GLM adjusted for age, gender and center was used to compare genotype groups in terms of metabolic syndrome criteria. Subjects receiving a treatment that may have affected the level of the variable were excluded from these analyses. Chi-square tests and logistic regression analyses were used to test the association between the various genotypes and metabolic syndrome. For logistic regression analyses, carriers of at least one minor allele were compared with subjects who were homozygous for the frequent allele (dominant model), while adjusting for age, gender and center. The threshold for statistical significance was set to 5%. Linkage disequilibrium between loci was tested using a log-likelihood-ratio test [29]. Disequilibrium was expressed in terms of normalized difference D' = D/D_max _or D/D_min _[30]. Haplotype frequencies were estimated using a stochastic version of the expectation-maximization algorithm, as implemented in Thesias software [31,32]. Differences in haplotype frequencies when comparing groups of subjects with or without metabolic syndrome were examined using a log-likelihood ratio statistic test, which was computed from the haplotype frequency log-likelihoods for each of the two groups estimated in Thesias.

## Results

Table 1 shows the biological and clinical characteristics of the subjects with (n = 932) and without (n = 2206) metabolic syndrome as defined by NCEP III. As expected, body mass index, waist girth, triglyceridemia, cholesterolemia, glycemia, systolic and diastolic blood pressure were higher and HDL-cholesterol levels were lower in subjects with metabolic syndrome than in subjects without metabolic syndrome (all p < 0.0001). Subjects with metabolic syndrome were less physically active and had spent less time in education than those without metabolic syndrome. The proportion of smokers was lower in the metabolic syndrome group.

**Table 1:** Anthropometric, clinical and biological characteristics of subjects with or without metabolic syndrome

	Without metabolic syndrome	With metabolic syndrome	*p*
n	2206	932	
age (y)	49.4 ± 8.4	54.2 ± 8.1	*<0.0001*
men	1044 (47.3)	553 (59.3)	*<0.0001*
BMI (kg/m^2^)	24.8 ± 3.6	29.9 ± 4.7	*<0.0001*
Waist (cm)	85.3 ± 11.3	101.5 ± 11.6	*<0.0001*
Cholesterol (mmol/l)	5.8 ± 1.0	6.1 ± 1.1	*<0.0001*
Triglycerides (mmol/l)	1.00 ± 0.52	2.20 ± 2.32	*<0.0001*
HDL (mmol/l)	1.58 ± 0.43	1.19 ± 0.34	*<0.0001*
Glucose (mmol/l)	5.20 ± 0.76	5.95 ± 1.30	*<0.0001*
Diabetes (%)	2.4	19.5	*<0.0001*
SBP (mm Hg)	125.7 ± 15.8	140.6 ± 16.9	*<0.0001*
DBP (mm Hg)	79.0 ± 10.1	87.9 ± 11.2	*<0.0001*
			
Smoking			*0.032*
never	1069 (48.5)	432 (46.4)	
former	629 (28.5)	308 (33.0)	
current	508 (23.0)	192 (20.6)	
			
Physical activity			*<0.0001*
none	449 (20.4)	244 (26.3)	
light	1058 (48.0)	490 (52.7)	
intense	697(31.6)	195 (21.0)	
			
Alcohol intake			*0.0013*
Q1	760 (34.5)	303 (32.5)	
Q2	574 (26.0)	201 (21.6)	
Q3	535 (24.3)	241 (25.9)	
Q4	337 (15.2)	187 (20.0)	
			
Educational level			*<0.0001*
low	433 (19.6)	312 (33.5)	
intermediate	952 (43.2)	410 (44.0)	
high	821 (37.2)	210 (22.5)	

Values are means± SD or n (%).

BMI : body mass index ; HDL : high density lipoprotein ; SBP/DBP : systolic and diastolic blood pressure ; alcohol intake is in quartiles.

The *APOA5 *Ser19Trp,*APOA5 *-12,238T>C, *APOA4 *Thr347Ser, *APOC3 *-482C>T and *APOC3 *3238C>G genotypes did not deviate from Hardy-Weinberg equilibrium. Table 2 shows the linkage disequilibrium matrix in subjects without metabolic syndrome. Overall, the |D'| varied from 0.29 to 1. The *APOA*5 and *APOA4 *SNPs were in the same haplotype block (|D'|≥0.85); however, due to differences in minor allele frequencies, the r^2 ^values were almost nil. Within this block, both *APOA5 *-12,238T>C and *APOA4 *Thr347Ser SNPs were in negative disequilibrium with the *APOA5 *Ser19Trp SNP. Both *APOC3 *SNPs were within the same linkage disequilibrium block (D' = 0.91). Lastly, the *APOA4 *Thr347Ser SNP was in negative linkage disequilibrium with the *APOC3 *3238C>G SNP (D' = -0.92) and thus showed some overlap with the *APOC3 *haplotype block.

**Table 2:** D' and r^2 ^values for linkage disequilibrium among polymorphisms in subjects without metabolic syndrome

	*APOA5 Ser19Trp*	*APOA5 -12,238 T>C*	*APOA4 Thr347Ser*	*APOC3 -482 C>T*	*APOC3 3238 C>G*
*APOA5 Ser19Trp*	-	*-0.85*	*-1*	*-0.73*	*-0.41*
*APOA5 -12,238 T>C*	*0.02*	-	*0.95*	*0.29*	*-0.58*
*APOA4 Thr347Ser*	*0.02*	*0.40*	-	*0.45*	*-0.92*
*APOC3 -482 C>T*	*0.01*	*0.06*	*0.13*	-	*0.91*
*APOC3 3238 C>G*	*0.00*	*0.02*	*0.02*	*0.23*	-

D' upper right corner ; r^2 ^lower left corner

Table 3 shows the metabolic syndrome-related anthropometric, biological and clinical parameters in subjects without metabolic syndrome and as a function of the 5 polymorphisms. Multivariate analyses (adjusted for age, gender and center) revealed that mean triglyceride levels were significantly higher in *APOA5 *Trp19 (p < 0.0001; p < 0.0001 in a dominant model),*APOC3 *-482T (p = 0.04 in a dominant model) and *APOC3 *3238G (p < 0.0001; p < 0.0001 in a dominant model) allele carriers. Conversely, mean triglycerides were lower in *APOA5 *-12,238C (p < 0.01; p = 0.003 in a dominant model), *APOA4 *Ser347 (p = 0.04 in a dominant model) allele carriers than in non-carriers. The mean HDL-cholesterol value was significantly higher in *APOA5 *-12,238C (p = 0.04; p = 0.02 in a dominant model) allele carriers than in non-carriers. Systolic and diastolic blood pressures were higher in *APOA5 *Trp19 carriers (p = 0.03 in a dominant model) and systolic blood pressure was lower in *APOA4 *Ser347 carriers (p = 0.03 in a dominant model). There were no other statistically significant associations for any of the SNPs. Further adjustment for physical activity, alcohol consumption, smoking habits and educational level yielded similar results (data not shown). These associations were similarly observed in all three centers (i.e. there was no significant interaction with geographical area).

**Table 3:** Mean levels of metabolic syndrome-related anthropometric, biological and clinical parameters in subjects without metabolic syndrome according to the 5 polymorphisms

***APOA5 Ser19Trp***	**Ser19Ser**	**Ser19Trp**	**Trp19Trp**	***p***	***p^a^***	***p^*b*^***
Waist (cm)	85 ± 11	84 ± 11	81 ± 8	*0.49*	*0.23*	*0.77*
TG (mmol/l) ^d^	1.26 ± 1.1	1.59 ± 2.4	2.46 ± 2.3	*<0.0001 *^*c*^	*<0.0001 *^*c*^	*0.07 *^*c*^
HDL (mmol/l) ^d^	1.48 ± 0.4	1.49 ± 0.5	1.2 ± 0.5	*0.56*	*0.78*	*0.59*
Glucose (mmol/l) ^e^	5.4 ± 1	5.4 ± 0.8	5.5 ± 0.4	*0.72*	*0.43*	*0.83*
SBP (mm Hg) ^f^	129 ± 17	131 ± 18	135 ± 13	*0.081*	*0.03*	*0.47*
DBP (mm Hg) ^f^	81 ± 11	82 ± 10	84 ± 10	*0.082*	*0.03*	*0.39*
						
***APOA5 -12,238 T>C***	**TT**	**TC**	**CC**			
Waist (cm)	85 ± 11	85 ± 11	85 ± 11	*0.49*	*0.23*	*0.15*
TG (mmol/l) ^d^	1.31 ± 1.0	1.29 ± 1.4	1.32 ± 1.9	*0.01 *^*c*^	*0.003 *^*c*^	*0.08 *^*c*^
HDL (mmol/l) ^d^	1.47 ± 0.4	1.49 ± 0.4	1.52 ± 0.5	*0.04*	*0.02*	*0.10*
Glucose (mmol/l) ^e^	5.4 ± 1	5.4 ± 1.1	5.3 ± 0.8	*0.31*	*0.52*	*0.15*
SBP (mm Hg) ^f^	129 ± 17	129 ± 18	129 ± 17	*0.24*	*0.11*	*0.30*
DBP (mm Hg) ^f^	81 ± 11	81 ± 11	81 ± 10	*0.79*	*0.54*	*0.49*
						
***APOA4 Thr347Ser***	**Thr347Thr**	**Thr347Ser**	**Ser347Ser**			
Waist (cm)	85 ± 11	86 ± 11	86 ± 11	*0.64*	*0.71*	*0.39*
TG (mmol/l) ^d^	1.31 ± 1	1.30 ± 1.8	1.22 ± 0.8	*0.11 *^*c*^	*0.04 *^*c*^	*0.69 *^*c*^
HDL (mmol/l) ^d^	1.48 ± 0.4	1.49 ± 0.4	1.55 ± 0.5	*0.24*	*0.24*	*0.09*
Glucose (mmol/l) ^e^	5.4 ± 1.1	5.4 ± 0.9	5.2 ± 0.7	*0.44*	*0.63*	*0.20*
SBP (mm Hg) ^f^	130 ± 18	129 ± 17	127 ± 16	*0.09*	*0.03*	*0.82*
DBP (mm Hg) ^f^	81 ± 11	81 ± 11	81 ± 10	*0.92*	*0.83*	*0.84*
						
***APOC3 -482 C>T***	**CC**	**CT**	**TT**			
Waist (cm)	85 ± 11	85 ± 11	85 ± 11	*0.75*	*0.78*	*0.49*
TG (mmol/l) ^d^	1.27 ± 1	1.34 ± 1	1.33 ± 1	*0.09 *^*c*^	*0.04 *^*c*^	*0.07 *^*c*^
HDL (mmol/l) ^d^	1.47 ± 0.4	1.49 ± 0.4	1.51 ± 0.4	*0.68*	*0.54*	*0.66*
Glucose (mmol/l) ^e^	5.4 ± 1	5.4 ± 1	5.4 ± 0.8	*0.93*	*0.78*	*0.87*
SBP (mm Hg) ^f^	129 ± 17	129 ± 18	128 ± 17	*0.47*	*0.45*	*0.28*
DBP (mm Hg) ^f^	81 ± 10	81 ± 11	81 ± 11	*0.93*	*0.72*	*0.85*
						
***APOC3 3238 C>G***	**CC**	**CG**	**GG**			
Waist (cm)	85 ± 11	86 ± 11	84 ± 15	*0.25*	*0.1*	*0.66*
TG (mmol/l) ^d^	1.27 ± 1.4	1.44 ± 1.2	1.56 ± 1.2	*<0.0001 *^*c*^	*<0.0001 *^*c*^	*0.05 *^*c*^
HDL (mmol/l) ^d^	1.49 ± 0.4	1.47 ± 0.4	1.43 ± 0.4	*0.15*	*0.6*	*0.29*
Glucose (mmol/l) ^e^	5.4 ± 1	5.4 ± 0.8	5.5 ± 0.6	*0.55*	*0.35*	*0.79*
SBP (mm Hg) ^f^	129 ± 17	129 ± 17	134 ± 21	*0.19*	*0.52*	*0.28*
DBP (mm Hg) ^f^	81 ± 11	81 ± 11	84 ± 12	*0.12*	*0.37*	*0.21*

p values ajusted on age, gender and center ; p^a ^and p^b ^values for a dominant and recessive models, respectively; ^c^analyses on log-transformed data ; BMI : body mass index ; TG : triglycerides ; HDL : high density lipoprotein ; SBP/DBP : systolic and diastolic blood pressure ; Exclusion of subjects treated : ^d ^with fibrates, ^e ^oral antidiabetic or insulin, ^f ^BP lowering therapy.

In order to assess the contribution of the genetic variability to plasma triglyceride levels, haplotype analyses were performed in subjects without metabolic syndrome using the 5 SNPs in the following order: *APOA5 *(Ser(C)19Trp(G), -12,238T>C),*APOA4 *(Thr(A)347Ser(T)) and *APOC3 *(-482C>T, 3238C>G). Eight haplotypes had an estimated frequency of above 1% (accounting for more than 97% of the existing haplotypes) and were used in the analyses (Table 4). The overall association between haplotypes and triglyceride levels was highly significant (p < 4 × 10^-8^). By reference to the most common haplotype (CTACC; estimated frequency: 0.45), two haplotypes were significantly associated with raised plasma triglycerides. The GTACC haplotype (estimated frequency: 0.05) that carried the *APOA5 *Trp19 allele on the common background was associated with a 30% increase in triglyceride levels (p < 10^-6^). The other haplotype (CTATG) carried both the *APOC3 *-482T and 3238G minor alleles on the common genetic background (estimated frequency: 0.08) and was also associated with a 30% increase in triglyceride levels (p < 10^-6^).

**Table 4:** Haplotype frequencies and delta values of mean triglycerides in subjects without metabolic syndrome

Haplotype	Frequency	Δ TG	*p*
CTACC	0.454	reference	-
CCACC	0.130	0.037	0.13
CCTTC	0.118	0.003	0.93
CTATG	0.079	0.165	<10^-6^
CCTCC	0.075	-0.004	0.92
GTACC	0.050	0.165	<10^-6^
CTATC	0.043	-0.021	0.71
CCATC	0.022	-0.013	0.83

Only haplotypes with a frequency above 1% are represented. Order of the SNPS: *APOA5 *(Ser(C)19Trp(G), 12,238T>C), *APOA4 *(Thr(A)347Ser(T)) and *APOC3 *(-482C>T, 3238C>G). p values adjusted for age, gender and center.

Table 5 shows the genotype distribution of the 5 SNPs in subjects with or without metabolic syndrome. The two groups of subjects differed in terms of the genotype distribution of *APOA5 *-12,238T>C (p = 0.026) and *APOA4 *Thr347Ser (p = 0.034) SNPs. The adjusted odds ratio (OR) and 95% confidence interval (CI) for metabolic syndrome in carriers of at least one minor allele (compared with subjects homozygous for the frequent allele) are presented in Table 6. The *APOA5 *Trp19 allele conferred an increased risk of metabolic syndrome (p = 0.03). In contrast, the *APOA5 *-12,238C (p = 0.01) and the *APOA4 *Ser347 (p = 0.04) alleles were associated with a lower risk of metabolic syndrome. Adjustment for plasma triglycerides but not for other metabolic syndrome criteria (i.e. waist girth, HDL, glycemia and systolic blood pressure) abolished these associations. Lastly, there was no significant association between the *APOC3 *-482C>T and 3238C>G polymorphisms and metabolic syndrome. Using the IDF criteria for metabolic syndrome, the ORs [95%CI] for metabolic syndrome were similar in magnitude (data not shown).

**Table 5:** Genotype distributions in subjects with or without metabolic syndrome (MS)

SNP				*p*
*APOA5 Ser19Trp*	Ser19Ser	Ser19Trp	Trp19Trp	
Without MS	1960 (88.8)	241 (10.9)	5 (0.2)	*0.10*
With MS	807 (86.6)	120 (12.9)	5 (0.5)	
*APOA5 -12,238 T>C*	TT	CT	CC	
Without MS	912 (41.3)	995 (45.1)	299 (13.5)	*0.026*
With MS	413 (44.3)	424 (45.5)	95 (10.2)	
*APOA4 Thr347Ser*	Thr347Thr	Thr347Ser	Ser347Ser	
Without MS	1412 (64.0)	702 (31.8)	92 (4.2)	*0.034*
With MS	627 (67.3)	282 (30.3)	23 (2.5)	
*APOC3 -482 C>T*	CC	CT	TT	
Without MS	1148 (52.0)	894 (40.5)	164 (7.4)	*0.26*
With MS	508 (54.5)	349 (37.4)	75 (8.0)	
*APOC3 3238 C>G*	CC	CG	GG	
Without MS	1799 (81.5)	388 (17.6)	19 (0.86)	*0.71*
With MS	750 (80.5)	172 (18.4)	10 (1.1)	

Values are number (%).

**Table 6:** Odds ratios for metabolic syndrome in carriers of the minor allele

SNP	OR	[95% CI]	*p*
*APOA5 Ser19Trp*	1.30	[1.03–1.66]	*0.03*
*APOA5 -12,238 T>C*	0.81	[0.69–0.95]	*0.01*
*APOA4 Thr347Ser*	0.84	[0.70–0.99]	*0.04*
*APOC3 -482 C>T*	0.89	[0.76–1.06]	*0.17*
*APOC3 3238 C>G*	1.03	[0.84–1.26]	*0.76*

The reference groups were homozygous subjects for the frequent allele. Odds ratios and p values adjusted for age, gender and center.

Haplotype analyses using the 5 SNPs were performed to assess the relationship with metabolic syndrome. The overall association between the haplotypes and metabolic syndrome was statistically significant (p = 0.011). With respect to the most common haplotype (CTACC), 2 haplotypes were significantly associated with metabolic syndrome. The GTACC haplotype (carrying the *APOA5 *Trp19 allele on the common background) was associated with a 33% greater risk of metabolic syndrome (p < 0.005). The other haplotype (CCTTC, carrying the *APOA5 *12,238C, the *APOA4 *Ser347 and the *APOC3 *-482T alleles) was associated with a 26% reduction in metabolic syndrome risk (p = 0.03). Since the *APOC3 *and *APOA5 *SNPs are in different haplotype blocks, the association with metabolic syndrome was tested for the two blocks independently. Using only the SNPs of *APO5 *(Ser19Trp, -12,238T>C) and *APOA4 *Thr347Ser, we found 4 possible haplotypes with a frequency of above 1%. When compared with the common haplotype (CTA; estimated frequency: 0.58), the GTA (estimated frequency 0.05) and the CCT (estimated frequency 0.19) haplotypes were respectively associated with a 33% greater risk (p < 0.02) and a 17% lower risk (p = 0.034) of metabolic syndrome. Both associations disappeared after adjustment for triglyceride levels. In contrast, haplotype analyses with the two *APOC3 *SNPs did not reveal any significant association with metabolic syndrome.

## Discussion

The goal of the present study was to assess the contribution of SNPs in the APOA5/A4/C3/A1 cluster to the risk of metabolic syndrome. Our results showed that the *APOA5 *Trp19 allele was associated with an increased risk of metabolic syndrome and that this association could possibly be explained by an increase in plasma triglyceride levels. Furthermore, the *APOA5 *-12,238C and *APOA4 *Ser347 alleles were associated with a lower risk of metabolic syndrome – possibly due to their negative linkage disequilibrium with the *APOA5 *Trp19 allele. In contrast, the *APOC3 *-482C>T and 3238C>G SNPs did not appear to be related to metabolic syndrome in this French sample.

All 5 SNPs of the cluster tested in this study were associated with triglyceride levels. Compared with the most common haplotype, haplotypes that carried either the *APOA5 *Trp19 allele only or both the *APOC3 *-482T and *APOC3 *3238G alleles were found to be independently associated with elevated triglycerides. These results are consistent with earlier studies in different population samples that reported similar linkage disequilibrium patterns and associations with triglycerides (driven by the *APOA5 *Trp19 and *APOC3 *-482T alleles [20]). In addition to elevated triglyceride levels, the *APOA5 *Trp19 and *APOA4 *Thr347 alleles were associated with higher blood pressure. A direct effect of APOA5 or APOA4 on blood pressure regulation is unlikely. In contrast, there is experimental evidence to suggest that chronic hypertriglyceridemia leads to endothelium dysfunction, which is associated with an impaired response to vasodilator stimulation [33] and a subsequent decrease in nitric oxide availability [34] – phenomena which may result in increased blood pressure.

The *APOA5 *Trp19 allele increased the odds ratio for metabolic syndrome, whereas the *APOA5 *-12,238C and *APOA4 *Ser347 alleles decreased it. Other studies have reported associations between *APOA5 *SNPs and metabolic syndrome [8-12]. Our results extend this observation to a different European population sample. The reduced risk of metabolic syndrome observed in carriers of *APOA5 *-12,238C and *APOA4 *Ser347 alleles merely reflected the negative linkage disequilibrium existing between these alleles and the *APOA5 *Trp19 allele. Several lines of evidence suggest that the association with metabolic syndrome is mediated by triglycerides. Firstly, the *APOA5 *Trp19 allele is associated strongly and independently with triglyceride levels but far less so with blood pressure. Secondly, adjustment for triglycerides (but not for other metabolic syndrome-related criteria) abolished the associations between *APOA5 *or *APOA4 *SNPs and the risk of metabolic syndrome. Overall, this suggests that *APOA5 *and *APOA4 *genetic variability affected susceptibility to metabolic syndrome.

In contrast, the *APOC3 *-482C>T and *APOC3 *3238C>G SNPs did not significantly affect the risk of metabolic syndrome in this sample of French origin, despite significant association with triglyceride level. These SNPs do not belong to the same haplotype as the *APOA5 *Ser19Trp SNP and have an independent effect on plasma triglycerides, which may explain the difference vis-à-vis APOA5. Furthermore and in contrast to the *APOA5 *Ser19Trp SNP, the *APOC3 *SNPs were not associated with another component of metabolic syndrome (such as blood pressure) which could explain this difference. Lastly, the prevalence of hypertriglyceridemia was higher in carriers of the *APOA5 *Trp19 allele (35%) than in carriers of the *APOC3 *-482T (23%) or APOC3 3238G (22%) alleles and thus increased the probability of fulfilling the definition of metabolic syndrome.

This study has both strengths and limitations. It was performed on a representative sample of the French population and therefore avoided potential selection bias for patients and controls. There were more than 900 subjects with metabolic syndrome, which yields enough statistical power to detect an OR of 1.3 for an allele frequency of 10% at α = 0.05 and β = 90%. Despite this large number of subjects with metabolic syndrome, the statistical power of the study was still not sufficient to account for possible type 1 error due to multiple testing. Therefore, corrections for multiple testing were not applied in the present analysis. In our sample, subjects without metabolic syndrome were, on average, 5 years younger than those with metabolic syndrome. Since metabolic syndrome prevalence increases with age, this may possibly confound (underestimate) the odds ratio associated with the SNPs. However, all our analyses were adjusted for age, in order to account for a possible impact of the latter parameter on the association. Since only a few SNPs per gene were studied, we cannot completely rule out the possibility that other SNPs in different gene loci or specific haplotypes can also contribute to genetic susceptibility to metabolic syndrome. Indeed, other studies have reported associations between *APOA5 *SNPs and metabolic syndrome [8-12]. Lastly, we used the NCEP working definition of metabolic syndrome; however, additional analyses using the IDF consensus definition generated very similar conclusions and suggested that choice of the definition of metabolic syndrome does not affect the relationship between metabolic syndrome and the SNPs studied here.

## Conclusion

Over the past decades, genetic variability in the APOA5/A4/C3/A1 cluster has been associated (to varying extents) with variability in plasma lipid, apolipoprotein and lipoprotein levels and with an increased risk of cardiovascular disease. The results of the present study further suggest that the association of SNPs in this cluster with metabolic syndrome may be explained by the propensity of plasma triglycerides to increase in *APOA5 *Trp19 allele carriers.

## Competing interests

The authors declare that they have no competing interests.

## Authors' contributions

DC, AW, PD, JBR, DA, AB, JF and PA participated in the design and coordination of the study. JD and AM carried out the molecular genetic studies, performed the statistical analyses, wrote the paper. All authors read and approved the final manuscript.

## Pre-publication history

The pre-publication history for this paper can be accessed here:


